# Customised-sampling approach for pipe failure prediction in water distribution networks

**DOI:** 10.1038/s41598-024-69109-9

**Published:** 2024-08-06

**Authors:** Milad Latifi, Ramiz Beig Zali, Akbar A. Javadi, Raziyeh Farmani

**Affiliations:** Centre for Water Systems, University of Exeter, Exeter, UK

**Keywords:** Failure prediction in pipes, Water distribution networks, Machine learning, Imbalance class data, Under-sampling, Over-sampling, Class weighting, Engineering, Civil engineering

## Abstract

This paper presents a new methodology for addressing imbalanced class data for failure prediction in Water Distribution Networks (WDNs). The proposed methodology relies on existing approaches including under-sampling, over-sampling, and class weighting as primary strategies. These techniques aim to treat the imbalanced datasets by adjusting the representation of minority and majority classes. Under-sampling reduces data in the majority class, over-sampling adds data to the minority class, and class weighting assigns unequal weights based on class counts to balance the influence of each class during machine learning (ML) model training. In this paper, the mentioned approaches were used at levels other than “balance point” to construct pipe failure prediction models for a WDN with highly imbalanced data. F1-score, and AUC–ROC, were selected to evaluate model performance. Results revealed that under-sampling above the balance point yields the highest F1-score, while over-sampling below the balance point achieves optimal results. Employing class weights during training and prediction emphasises the efficacy of lower weights than the balance. Combining under-sampling and over-sampling to the same ratio for both majority and minority classes showed limited improvement. However, a more effective predictive model emerged when over-sampling the minority class and under-sampling the majority class to different ratios, followed by applying class weights to balance data.

## Introduction

Water distribution networks (WDNs) are intricate systems of pipes, valves, and storage facilities designed to supply water to communities, industries, and households safely. These systems are responsible for delivering clean and potable water, which is essential for various purposes, including drinking, cooking, sanitation, and industrial processes. WDNs are important in sustaining human life and economic activities. However, they often encounter various challenges, with pipe failures being a significant concern.

Pipe failures within WDNs can be attributed to several factors, including ageing infrastructure, corrosion, material defects, external forces, operational conditions, and inadequate maintenance. These issues among others often lead to significant costs, water loss, and potential risks to public health and environment. Consequently, maintenance of these networks becomes a critical aspect of ensuring the efficient and reliable operation of the system. It encompasses a range of activities to preserve the functionality, integrity, and performance of assets throughout their lifespan.

Because the deterioration rates are linked to the condition and operation of the WDN, it is essential to determine which assets require prompt maintenance^1^. Current approaches to managing and maintaining the assets are mainly categorised into tactical and strategic maintenance of water infrastructures. Tactical rehabilitation involves swiftly addressing assets in need, while strategic rehabilitation focuses on preventing long-term failures through comprehensive, extended-term repair. This categorisation aids in selecting a systematic and efficient method for prioritising maintenance.

Water industry is currently shifting away from traditional reactive maintenance approaches, opting instead for more predictive and proactive solutions^2^. Reactive maintenance includes the monitoring, repairing, and replacing pipes in response to failures^3^. However, a more proactive approach involves modelling the deterioration of the network and assessing the risk of failure in its components to prevent bursts^3^. Through the utilisation of data-driven methodologies and predictive analysis, it becomes possible to evaluate the reliability of WDNs and anticipate the occurrence of failures. Consequently, this enables the implementation of predictive maintenance as a component of proactive one.

Data-driven pipe failure prediction tools can be divided into three main categories: deterministic, probabilistic, and machine learning (ML) methods^4^. Deterministic models use engineering principles to anticipate failure. These models employ mathematical equations and physical properties to analyse the behaviour of the WDN and determine the likelihood of failure^5^. However, deterministic models may not consider uncertainties and variations in the system, which can be a limitation, leading to limited accuracy in failure prediction^6^.

In order to address the limitations of deterministic models, probabilistic models incorporate uncertainties and variations by leveraging probability theory and statistical analysis. These models evaluate the probability of failure by analysing the distribution of failure events and estimating the likelihood of their occurrence^7^. Additionally, probabilistic models can handle randomness effectively and are valuable for predicting the time to next failure^7^.

Another way to predict pipe failures is through ML techniques, which utilise algorithms and computational models to identify patterns and make predictions based on data^8^. These models are proficient in analysing extensive datasets and identifying intricate relationships between variables to predict failures^9^. ML models can effectively manage uncertainties and non-linear relationships based on data, making them well-suited for complex systems like WDNs.

Availability of comprehensive and accurate data is crucial for making informed decisions and utilising data-driven methods^10^. However, the challenges of data shortage and class imbalance within WDN datasets present significant obstacles to developing an accurate failure prediction model. The issue of data shortage pertains to the limited availability of real-time or historical failure data. In the context of pipe failure prediction utilising ML, class imbalance refers to the uneven distribution of data into classes, for instance, the number of pipes which experienced failures are limited, which can negatively affect the performance of classifiers^11^.

Imbalance data occurs when the population of different classes are significantly uneven, meaning that one class is underrepresented in comparison with the others. In the context of classification in class imbalance, classes are usually grouped to majority and minority class. Majority is the class that has larger number of instances or samples in the dataset. Conversely, the minority class has fewer instances in the dataset. To calculate the majority and minority class ratios in a dataset, Eqs. (1 and 2) can be considered:

1 $$ Majority\,\,class\,\,ratio = \frac{Number\,\,of\,\,majority\,\,samples}{{Total\,\,number\,\,of\,\,samples}}, $$

2 $$ Minority\,\,class\,\,ratio = \frac{Number\,\,of\,\,\min ority\,\,samples}{{Total\,\,number\,\,of\,samples}}, $$

where, $Number\,\,of\,\,majority/\min ority\,\,samples$ refer to the count of instances belonging to majority/minority classes, respectively.

Imbalance class can pose challenges for ML models, as they may become biased towards the majority class, i.e., the trained model tends to classify the new instances in the majority class.

## Conventional approaches in dealing imbalance data

Diverse methodologies have been suggested to counter the challenge of class imbalance. These include over-sampling and under-sampling techniques, which aim to resolve the skewed distribution of classes^12^. For instance, random over-sampling (ROS) involves duplicating random samples from the minority class, whereas random under-sampling (RUS) removes samples from the majority class randomly^13^ to balance the data.

### Under-sampling

Under-sampling is a technique employed to tackle class imbalance in datasets by decreasing the number of instances in the majority class. The objective is to achieve a more equitable class distribution, thereby preventing biases in ML models. Positive aspects of under-sampling include improved computational efficiency due to reduced dataset size, facilitating faster model training and requiring less memory. A balanced dataset simplifies the model's learning task by avoiding the challenges associated with a heavily skewed class distribution, potentially resulting in enhanced generalisation and performance on the minority class^14^.

Despite these advantages, under-sampling has its drawbacks. Removing instances from the majority class may lead to a loss of valuable information, potentially discarding crucial patterns inherent in the majority class and impacting the model's generalisation capabilities. Under-sampling also heightens the risk of overfitting, especially when the original dataset is small because under-sampling keeps few instances from the majority class and the model may memorise rather than learn the patterns in the dataset. It is essential to acknowledge these trade-offs and carefully consider the specific characteristics of the dataset and problem domain when contemplating the use of under-sampling^15^.

#### Random under-sampling

Random under-sampling (RUS) is a technique employed to address class imbalance by randomly eliminating instances from the majority class until a more balanced class distribution is achieved. This technique uses a function for random selection. If *S* is a set of instances, and *n* is the desired number of instances to be randomly selected, the result can be denoted as $RandomSampler(S,n)$ as below:

3 $$ \begin{aligned} RandomSampler\;(S,n) & = \left\{ {s_{i} |s_{i} \, is \,a \,randomly\, selected \,instance \,from \,D_{original} } \right. \\ & \quad \left. {for\, i = 1,\,\,2,\,\, \ldots ,\,\,n} \right\}. \\ \end{aligned} $$

With this function in place, if $D_{original}$ is the original dataset, random under-sampling can be expressed as:

4 $$ D_{RUS} = D_{minority} \cup RandomSampler(D_{majority} ,\,\,n_{minority} ), $$

where, $D_{majority}$ and $D_{minority}$ are subsets of instances belonging to majority and minority class, respectively, $n_{minority}$ is the number of instances in minority class. The result is $D_{RUS}$, a dataset with balanced class distribution. In a general case, the second argument in $RandomSampler$ function can be selected to be higher or lower than the $n_{minority}$, allowing for the selection of instances from the majority class to be more or less than the number of minority class members.

#### Tomek links under-sampling

Tomek Links Under-sampling^16^ refers to pairs of instances—one from the majority class and the other from the minority class—that are in close proximity but belong to opposite classes. The purpose of identifying and eliminating instances involved in Tomek Links is to improve the separation between the majority and minority classes in the feature space. By removing instances from the majority class in each Tomek Link, the algorithm effectively increases the distance or space between the two classes. This process is intended to refine the decision boundary and alleviate the impact of noisy or borderline instances from the majority class on the classification model. The ultimate objective is to establish a more distinct separation between the classes, thereby facilitating the learning process for ML models when dealing with imbalanced data.

#### Near miss under-sampling

Near Miss Under-sampling is another algorithm that removes instances from the majority class that are close to instances from the minority class. There are rules and conditions under which instances from the majority class are considered “near” the instances from the minority class. Near Miss has three different approaches, each aim to remove majority samples to balance the classes. (1) For each minority class sample, NM-1 finds the *m* nearest majority class instances and removes majority samples near to the minority sample. (2) NM-2 is similar to NM-1 in removing instances but keeps *m* farthest majority samples, instead of nearest. (3) For each minority class sample *x*_*i*_ NM-3 finds the *m* farthest majority class instances as well as the *m* nearest majority instances.

The choice between approaches depends on the specific problem and the characteristics of the dataset. Near Miss algorithm uses distance metrics like Euclidean distance to find the nearest and farthest neighbours. This algorithm leverages distance calculations from minority class instances $x_{i}$ to majority class samples $x_{j}$, $D(x_{i} ,x_{j} )$, and selects based on shortest and farthest distances depending on the selected condition^17^.

### Over-sampling

Over-sampling is a technique employed to address class imbalance in datasets by increasing the number of instances in the minority class. The aim is to achieve a balanced class distribution, enabling ML models to learn from sufficient instances in the minority class. This approach offers several advantages, such as enhancing model performance by generating additional instances in the minority class. It ensures that the model has enough instances from both classes, thereby mitigating biases that may emerge when the training dataset is dominated by the majority class. In contrast to under-sampling, which involves removing instances from the majority class, over-sampling retains all instances from the majority class, preserving potentially valuable information that aids in capturing intricate relationships between data points by ML models.

Despite their benefits, over-sampling methods come with certain limitations. Over-sampling can result in overfitting, especially when duplicating or synthesising the same instances from the minority class. This tendency can cause the model to excel on the training data but perform poorly on unseen data. Additionally, when generating synthetic data for over-sampling, there is a risk of introducing noise into the dataset. Furthermore, the quality of the created synthetic data relies heavily on the original feature space and may not accurately represent the actual distribution of the minority class. In conclusion, experimentation and evaluation are essential for determining the effectiveness of over-sampling in addressing a specific problem^18^.

#### Random over-sampling

Random over-sampling (ROS) involves randomly duplicating instances from the minority class to address the class imbalance. Just like Eq. (3) for $RandomSampler$, ROS uses the same function for randomly selecting instances from $D_{minority}$ and duplicating them until reaching a desired level of over-sampling as:

5 $$ D_{ROS} = D_{majority} \cup RandomSampler(D_{minority} ,\,\,n_{majority} ). $$

In a general case, the number of instances selected from minority class could be less or more than that of majority class. In this research, the effect of variations in this number was studied^19^.

#### Synthetic Minority Over-sampling TEchnique (SMOTE)

The evolution of over-sampling techniques has introduced methods that go beyond the simple duplication of existing instances. One such technique is SMOTE, which, unlike random over-sampling, creates synthetic instances to augment the minority class^20^.

SMOTE operates by randomly selecting *k* nearest neighbours from the minority class, where *k* is a user-defined parameter. The technique then generates synthetic instances along the line segments that join these neighbours in the feature space. This process is repeated until the desired level of over-sampling is achieved^21^.

#### ADAptive SYNthetic sampling (ADASYN)

ADASYN stands as an extension of SMOTE, introducing adaptability to generating synthetic instances based on the density of instances within the feature space. The technique initiates by computing the class imbalance ratio, representing the proportion of majority to minority class instances in the dataset. For each minority data point, ADASYN dynamically determines the number of synthetic instances to generate. This determination involves a proportional relationship to the class imbalance ratio and takes into account the density distribution of instances in the vicinity of the current minority instance. In simpler terms, regions with lower instance density receive a higher number of synthetic instances. ADASYN proves particularly advantageous when dealing with datasets that display varying difficulty levels across different regions of the feature space^22^.

### Class weight

Class weighting is a technique to handle imbalance data in ML algorithms by assigning different weights to samples from various classes^23^. The standard way to train a model is to minimise an objective function, typically a loss function like cross-entropy for classification, over the entire dataset:

6 $$ Min\,\,\sum\limits_{i = 1}^{N} {Loss\left( {y_{i} ,\,\,\widehat{{y_{i} }}} \right)} , $$

where, $Loss\left( {y_{i} ,\,\,\widehat{{y_{i} }}} \right)$ calculates error between the observation $y_{i}$ and prediction $\widehat{{y_{i} }}$; and *N* is the number of samples. This technique first calculates the class imbalance ratio:

7 $$ Imbalance\,\,ratio = \frac{{n_{majority} }}{{n_{minority} }}, $$

in which, $n_{majority}$ and $n_{minority}$ are the number of instances in majority and minority class, respectively. By assigning majority class weight and minority class weight based on the imbalance ratio as:

8 $$ \left\{ \begin{gathered} Class\,\,Weight_{majority} = 1 \hfill \\ Class\,\,Weight_{minority} = Imbalance\,\,ratio \hfill \\ \end{gathered} \right.. $$

Class weighting assigns class weights in the loss function as:

9 $$ Total\,\,Loss = Class\,\,Weight_{majority} \,\sum {Loss_{majority} } + Class\,\,Weight_{minority} \,\sum {Loss_{minority} } . $$

By assigning higher class weights to the minority class, the model encourages to focus more on minimising the errors associated with the minority class during the learning phase^24^.

### Application of the conventional approaches in pipe condition assessment and failure prediction

When designing ML algorithms for failure prediction in the presence of class imbalance, most of the aforementioned methods were employed. Winkler et al. employed RUS in conjunction with a boosted decision tree, demonstrating superior performance in a real-world case study compared to other models^9^. Wijs et al. utilised SMOTE and class weighting techniques along with ML algorithms for pipe failure prediction, with the results indicating that RUS and weighting enhanced the predictive capabilities of the models^25^.

Robles-Velasco et al. combined ROS and RUS with the Artificial Neural Network (ANN) algorithm for balancing the minority and majority classes when developing a pipe failure prediction model and evaluated their effectiveness. They found out that under-sampling emphasises the accuracy of true positive rates, while over-sampling trains the system to predict both failures and non-failures with equal precision^26^. Dimas et al. applied SMOTE in conjunction with kNN and decision tree regression algorithms to develop a pipe failure prediction model to address class imbalance issues (6% failure in dataset). They discovered the necessity of over-sampling when building ML regressors in the presence of class imbalance^27^. Additionally, Fontecha et al. employed ROS, RUS, and SMOTE in a spatiotemporal analysis to predict failure risks of urban sewer systems. They crafted failure prediction models for different grid sizes. When dealing with high-resolution grids, they inevitably employed over-sampling techniques to achieve satisfactory outcomes^28^.

Liu et al. applied ROS, RUS, and SMOTE along with ML algorithms, such as Random Forest (RF) and logistic regression (LR), in a real case study in China for developing a pipe failure prediction model. The results suggested that RF utilising SMOTE exhibited more significant improvements compared to using ROS and RUS^29^. Beig Zali et al. encountered class imbalance issues due to shortage in historical failure data, while developing a pipe failure prediction model in a real case study. They utilised ROS, RUS, SMOTE, and class weight approaches in combination with ML algorithms, such as LR, RF, and Extreme Gradient Boosting (XGB). To construct a robust prediction model, they clustered the pipes based on their material, age, diameter, and length, integrating the *K*-means clustering method with domain knowledge expertise. This initiative enhanced the performance of the ML models in predicting the likelihood of pipe failures. Although their study proposed an approach to address the issue of class imbalance, among the traditional sampling techniques, class weighting demonstrated better results^30^.

Most of the research mentioned above have used over-sampling, under-sampling, and class weighting approaches in order to balance the minority and majority classes of data, based on an assumption that balanced data or weights better assist the ML model to train. In this paper, variable ratios were tested for under-sampling, over-sampling, and class weighting, to study their effects on the prediction capabilities of the trained ML models. In addition, combinations of under-sampling, over-sampling and class weight were examined to compare their results with individual methods.

## Case study

A WDN in the UK was selected to examine the proposed methodology. The case study comprises 43,000 km of pipes made from a wide range of materials, such as Polyethylene (PE), cast iron (CI), asbestos cement (AC), Polyvinyl Chloride (PV), ductile iron (DI), etc. The entire network consists of more than 937,000 pipes with varying lengths. The dataset includes inherent data about the pipes, such as diameter, length, age, material, etc. Failure data was available for a two-year period spanning from 2019 to 2020, with only a limited number of pipes experiencing multiple failures. To simplify the training process, all pipes were grouped into two binary categories: failed (with at least one failure) and not failed. 22,091 assets failures (2.36%) were recorded during the study period, resulting in an extremely imbalanced class data. Table 1 provides essential details about the WDN.

**Table 1:** Characteristics of the WDN.

Pipe feature	Range	Length (km)	Number of assets	Number of failures	Failure rate (failure/100 km/year)	Failure percentage (%)
Materials	PE	17,308.6	393,046	5490	14.64	1.40
	CI	8885.8	203,229	6867	35.67	3.38
	AC	6651.3	142,208	5545	38.48	3.90
	PV	6476.6	123,487	2829	20.16	2.29
	DI	1954.5	45,104	743	17.55	1.65
	Other	2121.6	30,494	617	13.42	2.02
Diameters	D < = 100 mm	14,781.2	347,685	8252	25.77	2.37
	100 < D < = 200	19,543.5	465,581	11,830	27.94	2.54
	D > 200	9073.6	124,300	2009	10.22	1.62
Ages	Age < = 20	9553.4	222,881	2736	13.22	1.23
	20 < Age < = 50	17,246.6	355,925	7245	19.39	2.04
	50 < Age < = 100	12,822.7	275,441	9559	34.41	3.47
	Age > 100	3775.6	83,321	2551	31.18	3.06
Total WDN length		43,398.4	937,566	22,091	23.49	2.36

**Figure 1:**
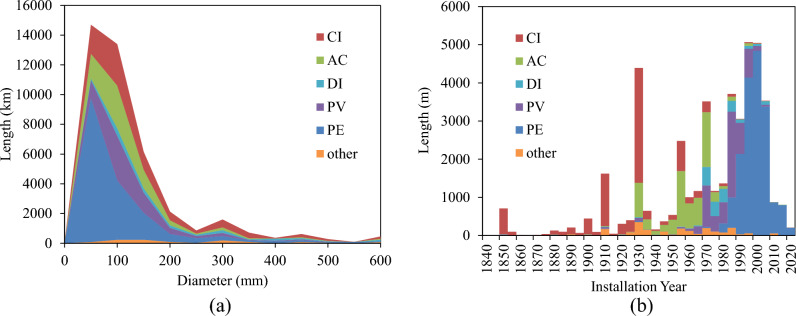
Distribution of (**a**) diameter; and (**b**) installation year, of pipes in the case study.

Figure 1a illustrates the diameter distribution of pipes in the WDN, revealing that 79% of the pipes fall within the diameter range of 50 to 150 mm. This figure also depicts the distribution of pipe materials, with 40% being composed of PE, 20% of CI, and 15% each for AC and PV. Figure 1b shows the age distribution for each pipe material. CI pipes are the oldest, while AC pipes were predominantly used from the 1930s to the 1970s. PV pipes gained popularity between the 1970s and 2000s, and PE pipes have been widely installed since the 1980s in the case study.

**Figure 2:**
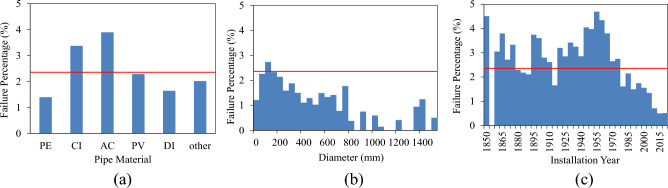
Distribution of failure percentage in various (**a**) pipe materials; (**b**) pipe diameters; and (**c**) installation years.

The failure percentage, i.e., the proportion of assets that have failed, serves as a metric for evaluating the presence of failure data in a dataset. A higher failure percentage indicates a greater availability of failure data, thereby enhancing the potential for effective training of a ML model. In the case study, only 2.36% of all assets experienced failure during the study period, which is relatively low for training a failure prediction model. Figure 2a illustrates the distribution of failure percentages for different groups of pipe materials. AC and CI exhibit failure percentages above the average (represented by the red line), specifically 3.9% and 3.4%, respectively. Figure 2b shows failure percentages across various pipe diameters. While most diameters have failure percentages below the average, pipes within the 50 to 150 mm range, constituting the majority, exhibit failure percentages around the mean (red line). Figure 2c displays failure percentages of pipes based on their installation year, revealing a noticeable decline in failure percentage for pipes installed since the 1960s. As anticipated, younger pipes are less likely to fail, whereas pipes installed during certain older periods surprisingly experienced minimal failures.

**Figure 3:**
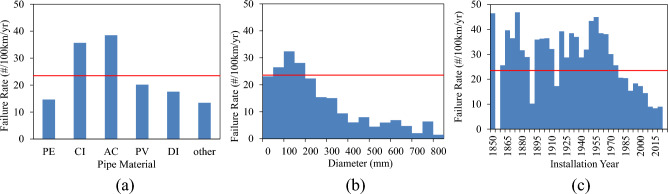
Distribution of failure rate in various (**a**) pipe materials; (**b**) pipe diameters; and (**c**) installation years.

The failure percentage evaluates the vulnerability of pipes, irrespective of their lengths. The failure rate, defined as the number of failures within a specific length of pipes over a designated time frame, offers a more precise assessment of the likelihood of failure in a group of pipes. In this study, the failure rate is computed as the number of failures per 100 km of pipes annually. The overall failure rate for the case study is 23.5 failures per 100 km per year. Figure 3a displays the failure rates for various pipe materials, with AC and CI pipes exhibiting the highest rates at 38.5 and 35.7 failures per 100 km per year, respectively. Figure 3b shows the distribution of failure rates across different pipe diameters, demonstrating a notable decrease in failure rate as pipe diameter increases. Figure 3c illustrates the failure rates for pipes categorised by their installation date, revealing a declining trend in the failure rate for pipes installed since 1960.

All three under-sampling methods mentioned in 2.1 were examined on the case study and the results showed that, among under-sampling approaches, RUS exhibited the most promising outcomes in the case study. Consequently, for the subsequent analysis, this method will be applied for under-sampling and, for the sake of simplicity, referred to as under-sampling. Moreover, among over-sampling approaches, SMOTE demonstrated superior results. As a result, moving forward, SMOTE will be employed to represent over-sampling methods and, for simplicity, referred to as over-sampling.

## Methodology

In this research, the effect of under-sampling, over-sampling, and class weighting with variable ratios and combining these methods on improvement of the performance of failure prediction models was investigated.

### Variable under-sampling

**Figure 4:**
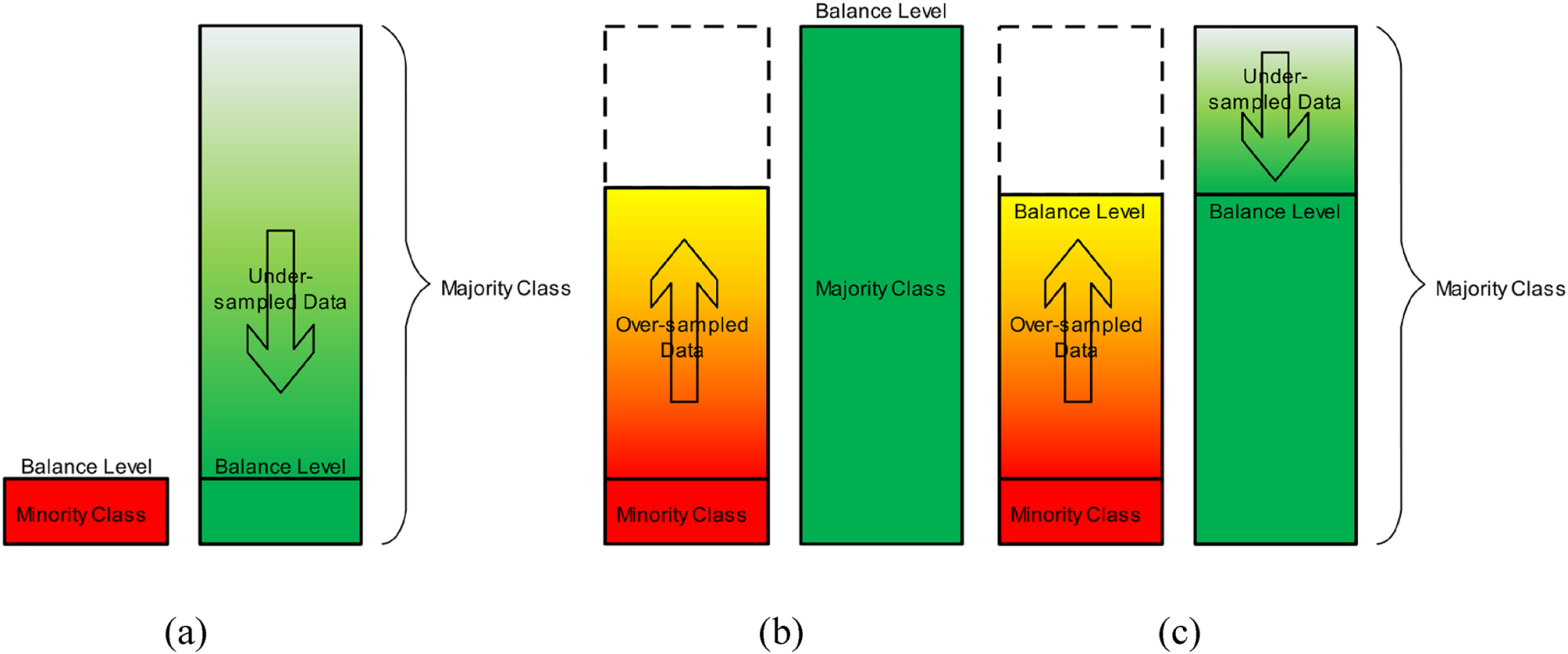
An illustration of (**a**) under-sampling; (**b**) over-sampling; and (**c**) combination of over- and under-sampling with variable ratios.

To investigate the impact of different ratios for under-sampling, the under-sampling function was implemented on the majority class data using various majority ratios (Eq. 1). The majority ratios initiate from the original majority ratio of the data, gradually decreasing until they match the minority ratio (resulting in a balanced distribution), and then continue decreasing further. Figure 4a provides a schematic representation of the gradual reduction in the number of majority class data.

### Variable over-sampling

To assess the influence of variable ratios for over-sampling, the function in Eq. (3) was implemented on the minority class. The minority class ratio was incremented from the original ratio in the dataset, gradually reaching the ratio of the majority class, and extending beyond that point. Figure 4b illustrates a schematic overview of the application of the variable over-sampling technique.

### Variable class weighting

This study investigates the impact of class weighting by assessing the effects of assigning varying weights to the minority class. Specifically, the weight of the majority class is always maintained as one. In contrast, the weight of the minority class is incremented from 1 to the balanced weight (Eq. 8) and further increased beyond this point. The expectation is to observe diverse prediction capabilities as the class weight changes.

### Combination of under-sampling and over-sampling to balance the data

In the subsequent stage, under-sampling and over-sampling functions were employed on the majority and minority class data, respectively, to achieve a balanced dataset. In this scenario, the number of instances in the majority class was under-sampled to a specific number, while concurrently, the number of instances in the minority class was over-sampled to match that same number. The impact of this combined under- and over-sampling was examined by varying the target ratio at each step. Figure 4c illustrates the outlined approach.

### Variable combination of under-sampling and over-sampling, and balancing with class weights

At this phase, the collective impact of under-sampling, over-sampling, and class weighting was under examination. Initially, the majority and minority classes underwent under-sampling and over-sampling, respectively, at distinct ratios. This implies that even after the initial step of under-sampling and over-sampling, the number of instances in each class remained imbalanced. Subsequently, in the following step, appropriate class weights were determined based on the new population of each class. Specifically, the weight of the majority class was maintained at one, while the weight for the minority class was calculated using Eq. (8).

### Failure prediction in pipes

Two ML models were constructed to predict pipe failures in the WDNs: (1) Random Forest (RF); and (2) Support Vector Machine (SVM). Latifi et al.^4^ reviewed the tree-based models and compared their performance with k-th Nearest Neighbours (kNN), Support Vector Machines (SVM), Artificial Neural Networks (ANN), among the others. They concluded that RF mostly outperforms other ML models in pipe condition assessment and failure prediction problems^4^. RF is an ensemble of decision trees, each trained on a randomly sampled subset of training data. This algorithm takes random bootstrap samples from training data, grows a decision tree, and selects the best split from a random subset of features. Predictions from $RF(x)$ for input *x* is made by aggregating predictions from the individual trees^31^:

10 $$ RF(x) = \arg \max \,\sum\limits_{i = 1}^{N} {I\left( {r_{t} (x_{i} ) = c} \right)} $$

where, $I\left( {} \right)$ is an indicator function; *c* is each class’s label; and *r*_*t*_ is the *t*-th decision tree that trained. Optimal trees for regression and classification can be ensembled by various metrics, e.g. Brier score and out-of-bag metric^32,33^.

SVM is an ML algorithm used for both classification and regression problems. It works by identifying a hyperplane that separates data into distinct classes, making it suitable for developing pipe failure prediction models^34^. For a dataset with *n* data points, where each data point has *m* features, and corresponds to class labels *y*, which are either 0 or 1 for binary classification, the hyperplane can be expressed as^35^:

11 $$ y = (w \cdot x) + b $$

where; *w* is the weight vector perpendicular to the hyperplane; *x* is the feature vector of a data point; and *b* is the bias term. If the value is positive, the data point is classified as 1 otherwise; it is classified as 0. This algorithm aims to find the values for *w* and *b* that maximise the distance between the hyperplane and nearest data point.

In this investigation, RF and SVM were employed to comprehend the intricate relationships between pipe features and forecast the likelihood of failure (LoF) for each pipe. For this purpose, the entire dataset was divided into a train set (70% of data) and a validation set (30% of data). The 70–30% split is a widely used convention that provides a good balance between having enough data for training the model and having sufficient data for validating its performance. This ensures that the model has adequate data to learn from while also providing a robust evaluation of its generalisation capability. The prediction model was trained leveraging the train set and tested against the validation set. Fivefold cross validation was carried out on the dataset to eliminate the effect of the part selected from dataset for training and validation. In the prediction phase, the model assigns a LoF to each pipe, representing the probability of failure in the pipe; a higher LoF indicates a greater vulnerability of the pipe.

By sorting the pipes based on their LoF, prioritisation can be established for rehabilitation, allowing the water utility to select a specific percentile (e.g., 1%) of the pipes for replacement. The number of failures predicted by the model when replacing a given percentile of the pipes can be utilised as an evaluation metric for failure prediction models. Since water utilities typically replace approximately 1% of their assets annually, replacing 5% of the assets might be associated with a short-term asset maintenance program. In comparison, a 20% pipe replacement may indicate of a long-term asset rehabilitation initiative.

Furthermore, by establishing a threshold for the LoF, pipes with LoF surpassing the threshold can be designated as failed, and vice versa. In such instances, True Positive (TP) and True Negative (TN) represent the number of pipes correctly predicted as failed and not failed, respectively. Additionally, False Negative (FN) and False Positive (FP) denote the number of pipes that are failed and not failed, respectively, but mispredicted. Precision, recall, and F1-score are defined as follows^36^:

12 $$ {\text{Precision = }}\frac{TP}{{(TP + FP)}} $$

13 $$ {\text{Recall = }}\frac{TP}{{(TP + FN)}} $$

14 $$ F{1 - }score{ = 2} \times \frac{{{\text{Precision}} \times {\text{Recall}}}}{{{\text{Precision}} + {\text{Recall}}}} $$

The Receiver Operating Characteristic (ROC) curve is a visual representation that illustrates the performance of a binary classifier model across various threshold values. This curve depicts the true positive rate ($TPR = TP/(TP + FP)$) against the false positive rate ($FPR = FP/(FP + TN)$) at each threshold setting. Another metric for assessing the performance of a binary classifier is the area under the ROC curve (AUC–ROC), which remains independent of the threshold. In this study, performance evaluation of the prediction model relies on *F*1-*score*, AUC–ROC, and the count of captured failures in 5% and 20% of pipe replacements^26,37^. 5% and 20% pipe replacement represent the tactical (short-term) and strategical (long-term) asset rehabilitation programs of the water utility, respectively.

## Results

### Variable under-sampling

**Figure 5:**
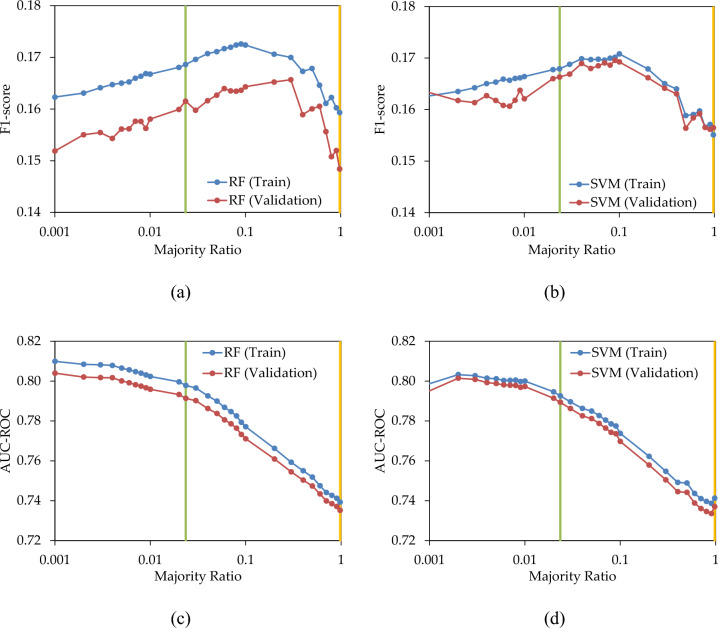
(**a**,**b**) F1-score; and (**c**,**d**) AUC–ROC; of RF and SVM prediction models with various under-sampling levels.

In the initial scenario, the majority class went through under-sampling, while the minority class remained untouched, in order to mitigate data imbalance. The original majority ratio of 0.9765 was reduced to achieve balance with the minority class at 0.0235, and subsequently lowered to 0.001. Figure 5a,b visually represent the alterations in the F1-score as the majority ratio decreases, transitioning from 0.9765 (amber line) to 0.0235 (green line—balanced) and then to 0.001 (minimum value). The F1-score in both the training and validation sets exhibits an increasing trend as the majority ratio decreases, peaking before declining again. Using RF, the maximum F1-scores for the training and validation sets are observed at majority ratios of 0.09 and 0.30, respectively. In SVM, the highest F1-scores occurred for the training and validation sets at majority ratios of 0.10 and 0.09, respectively. This illustration highlights that when under-sampling the majority class, the optimal F1-score does not necessarily coincide with a balanced ratio. In Fig. 5c,d, the changes in AUC–ROC under various under-sampling conditions are demonstrated for RF and SVM. Decreasing the majority ratio from its original value (0.9765) to a balanced value (0.0235) results in an increase in AUC–ROC, indicating an enhancement in the prediction model's performance. Further reductions in the majority ratio continue to improve AUC–ROC, albeit with a diminishing gradient. This pattern is observable in both the training and validation datasets.

**Figure 6:**
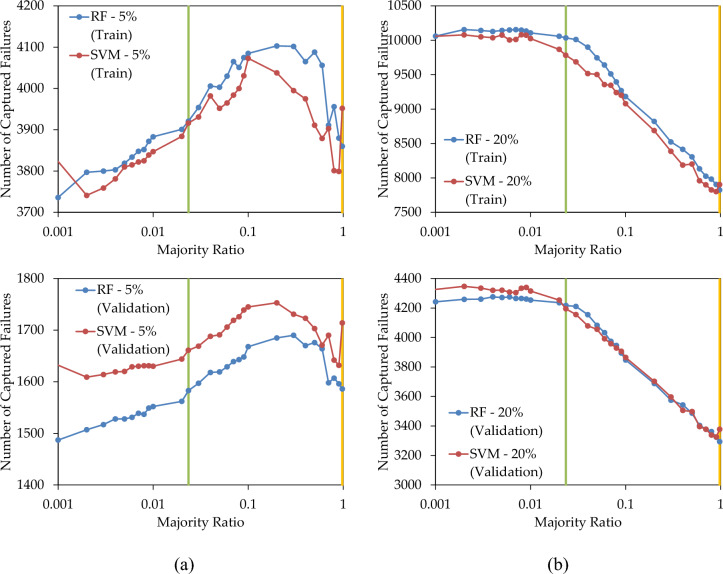
Number of failures captured by RF and SVM prediction models when replacing (**a**) 5%; and (**b**) 20%; of pipes, with various under-sampling levels.

In Fig. 6a, the variations in the number of failures detected by the prediction model are depicted as the majority ratio decreases, ranging from the original value of 0.9765 (amber lines) to the balanced value of 0.0235 (green lines) and the minimum value of 0.001. In both the training and validation sets of RF, a decrease in the majority ratio leads to an increase in the number of captured failures, reaching a peak at a majority ratio of 0.30, followed by a decline. In SVM, the same trend was observed with a peak at 0.1 and 0.2 for train and validation data sets, respectively. This figure illustrates that achieving the best prediction does not necessarily require under-sampling the majority class to achieve a balanced state. In other words, in this case, the optimal prediction occurred at a majority ratio greater than the balanced state. The similarity in the behaviour between the training and validation data suggests that the optimal majority ratio for the validation set can be inferred from the results of the training set.

Figure 6b portrays the variation in the number of captured failures as a function of the majority ratio, when replacing 20% of the high-LoF pipes. It is evident that decreasing the majority ratio enhances the prediction capability, resulting in more failures being predicted in both the training and validation sets. However, further reductions in the majority ratio beyond the balanced ratio yield only marginal improvements in the number of captured failures, suggesting that the balanced ratio is advantageous in addressing the imbalanced class dataset. Therefore, in this particular case study, under-sampling until achieving a balanced ratio is sufficient to yield a substantial number of failures, and further decreasing the majority ratio slightly improves the results. Furthermore, in Figs. 5, 6, the proportionality of train and validation results suggests that in each case, the optimal majority ratio of the validation dataset can be estimated through that of train dataset.

### Variable over-sampling

**Figure 7:**
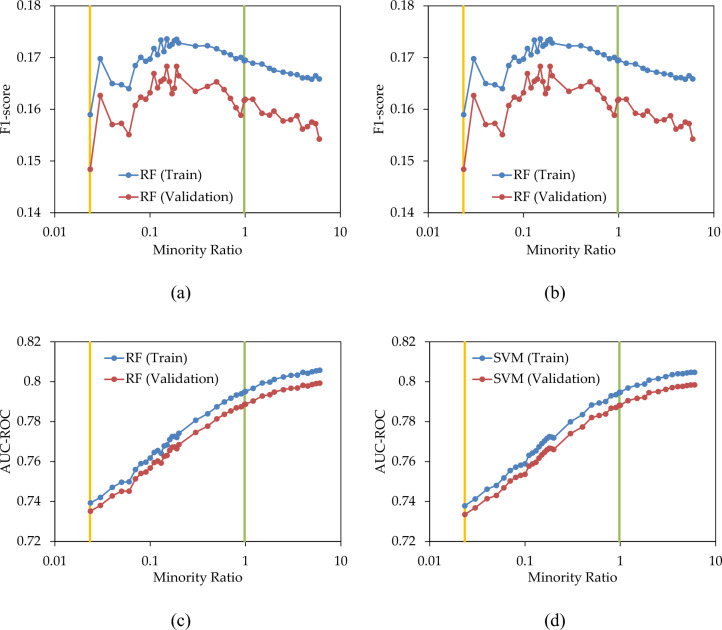
(**a**,**b**) F1-score; and (**c**,**d**) AUC–ROC; of RF and SVM prediction models with various over-sampling states.

In the second scenario, the approach to address imbalanced data involves over-sampling the minority dataset, while leaving the majority data unchanged. Consequently, the minority ratio increased from its original value (0.0235) to a balanced ratio (0.9765) and further increased beyond that, surpassing the population of the majority class. Figure 7a,b illustrate the fluctuations in F1-score as the minority ratio progresses from 0.0235 (amber line) to 0.9765 (green line) and then to 6 (an arbitrary maximum value). Minority ratios exceeding 0.9765 were chosen to observe the ultimate impact of over-sampling, specifically increasing the number of minority samples beyond that of the majority class. As the minority ratio increases, the F1-score shows improvement until it peaks at a minority ratio of 0.15 in both the training and validation sets, and both in RF and SVM. Beyond this ratio, increasing the minority ratio results in a decline in F1-score, indicating that over-sampling the minority class to a balanced ratio does not necessarily yield the optimal F1-score. In this case study, when the minority ratio was set at 0.15, the precision and recall values reached a point where their harmonic mean, F1-score, peaked. Due to the resemblance of train and validation graphs, the optimal minority ratio in validation set could be estimated from the results of train set.

Figure 7c,d demonstrate the variation in AUC–ROC as a function of the minority ratio. Over-sampling the minority class enhances AUC–ROC, even when the number of instances in the minority class exceeds that of the majority class.

**Figure 8:**
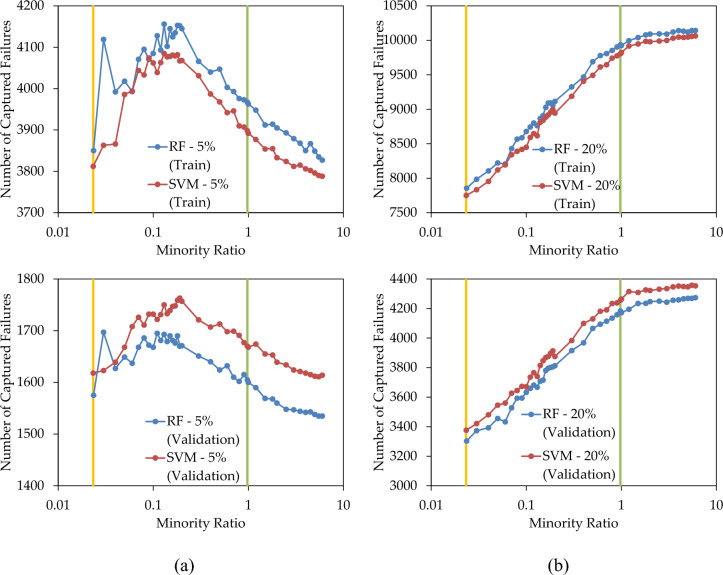
Number of failures captured by RF and SVM prediction models when replacing (**a**) 5%; and (**b**) 20%; of pipes, with various over-sampling levels.

Figure 8a illustrates the number of correctly predicted failures when 5% of pipes are replaced. Over-sampling the minority class resulted in an increased number of captured failures in both the training and validation sets. The maximum number of captured failures in the training set was observed when the minority ratio reached 0.13. Applying this ratio to the validation set led to a significant number of correctly predicted failures. Specifically, around 25% of failures were predicted by replacing only 5% of pipes. This implies that, in the context of this case study focusing on short-term pipe replacement, the minority ratio can be accurately estimated from the training set.

In Fig. 8b, the count of correctly captured failures when replacing 20% of pipes is presented. In both the training and validation sets, the number of accurate predictions increased with the over-sampling of the minority class, however, when the minority class population exceeded that of the majority class, the number of correctly predicted failures marginally increased. This implies that, in this case study, balanced ratio is a proper estimation for upper limit of over-sampling. Notably, 64% of failures in the validation set were correctly predicted by replacing only 20% of pipes.

### Variable class weighting

**Figure 9:**
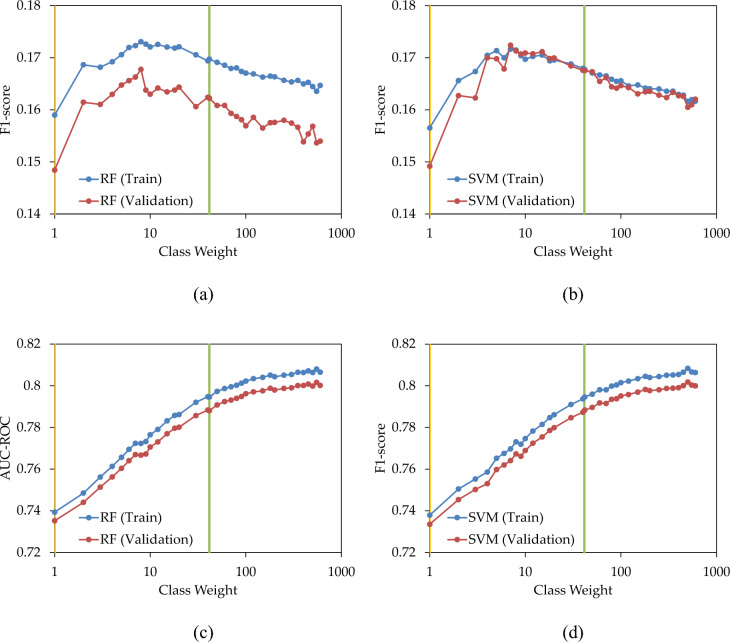
(**a**,**b**) F1-score; and (**c**,**d**) AUC–ROC; of the prediction models with various class weights.

In the third scenario, the weight of the minority class was adjusted to address the imbalanced class data. In this case study, the number of instances in the majority class is 41.5 times that of the minority class. To achieve class balance, weights ranging from one for the majority class to 41.5 for the minority class were assigned, extending up to 600 as an arbitrary maximum weight. Class weights greater than 41.5 were selected to explore the effect of assigning higher weights to minority class data compared to the majority class. Figure 9a,b display the variations in F1-score as the class weight changes for RF and SVM. With an increase in weight from one, the F1-score rose until it peaked at 8, observed in both the training and validation sets. Beyond this point, increasing the weight resulted in a nearly uniform decrease in F1-score. Notably, in this case study, the optimal F1-score occurred at a weight much lower than the traditional balanced weight (green line). Additionally, the optimal weight identified in the training set can be applied to achieve the highest F1-score in the validation set. Figure 9c,d depicts the changes in AUC–ROC for both the training and validation sets. In all cases, elevating the weight of the minority class enhanced AUC–ROC, even surpassing the balanced weight (green line). However, the rate of increase slowed down when the weight exceeded the balance value.

**Figure 10:**
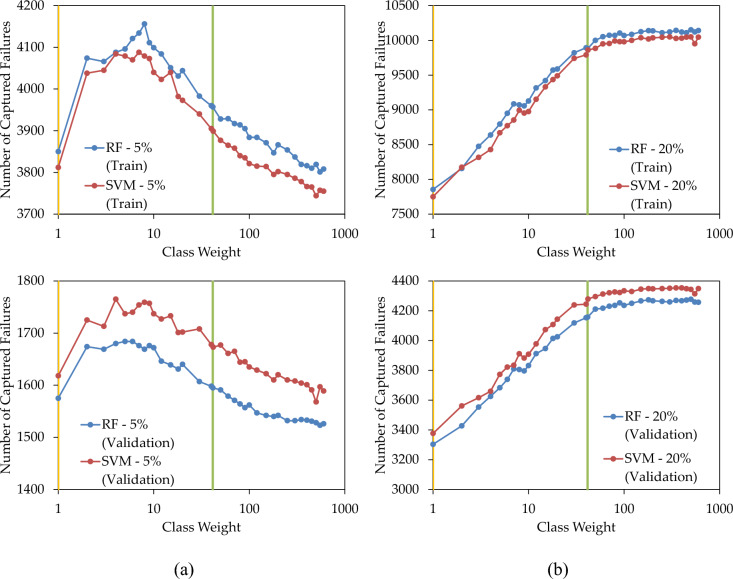
Number of failures captured by the prediction models when replacing (**a**) 5%; and (**b**) 20%; of pipes, with various over-sampling states.

Figure 10a illustrates the fluctuation in predicted failures when replacing 5% of pipes with the highest LoF. The increase in the weight of the minority class from one resulted in a corresponding increase in the number of accurate predictions. In training and validation sets of RF and SVM, the maximum number of correct predictions was observed at class weights between 4 and 8. Employing the optimal weight determined in the training set for the validation set continued to yield a high number of correct predictions. However, extending the class weight beyond these values led to suboptimal results.

In Fig. 10b, the count of correct failure predictions was depicted as a function of the class weight when replacing 20% of pipes. In both the training and validation sets, elevating the class weight enhanced the performance of the prediction model in identifying failures, albeit at a slower rate when surpassing the balancing weight. In Fig. 10, replacing 5% and 20% of pipes in the validation set resulted in the correct capture of 25% and 64% of failures, respectively.

### Combination of under-sampling and over-sampling to balance the data

**Figure 11:**
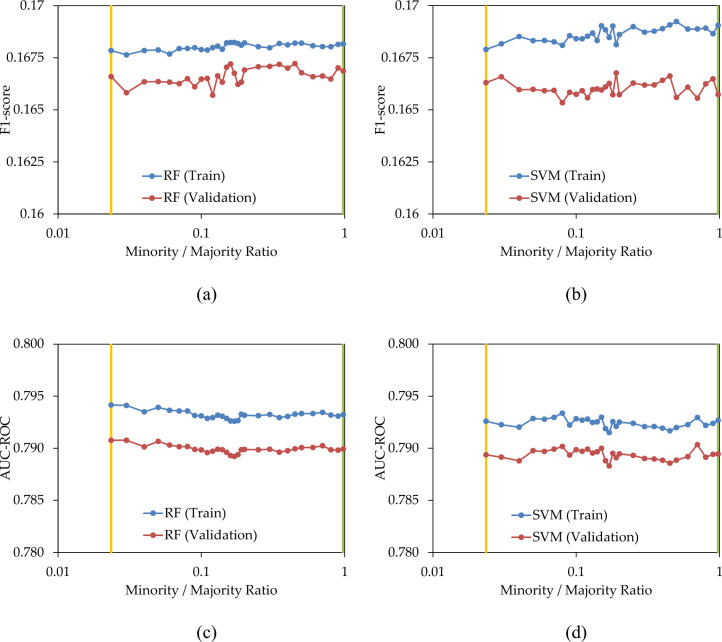
(**a**,**b**) F1-score; and (**b**,**d**) AUC–ROC; of the prediction models with combination of under-sampling and over-sampling.

In the subsequent scenario, both the minority and majority classes underwent treatment, specifically, under-sampling of the majority class and over-sampling of the minority class to achieve an equal population. Consequently, the minority/majority ratio increased from the original minority ratio of the dataset (represented by amber lines in Fig. 11) to the majority ratio of the dataset (illustrated by green lines in Fig. 11). Figure 11a,b reveal that the incremental increase in the ratio does not result in a noticeable change in the F1-score of the models, either in the training set or the validation set. Similarly, Fig. 11c,d display a slight change in AUC–ROC as the ratio increased from 0.0235 to 0.9765. In summary, the concurrent balancing of both minority and majority classes does not appear to enhance the predictive performance.

**Figure 12:**
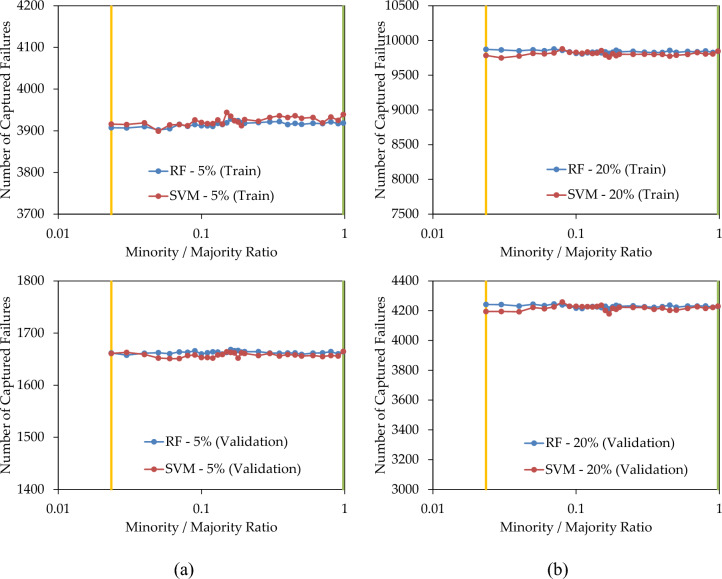
Number of failures captured by the prediction models when replacing (**a**) 5%; and (**b**) 20%; of pipes, with combination of under-sampling and over-sampling.

Figure 12a,b depict the count of failures correctly identified by the prediction model when replacing 5% and 20% of the pipes, respectively. In both these figures, the simultaneous over-sampling and under-sampling of the minority and majority classes to equal levels did not result in a significant improvement in the number of captured failures, thus supporting the conclusion drawn from Fig. 11.

### Variable combination of under-sampling and over-sampling, and balancing with class weights

**Figure 13:**
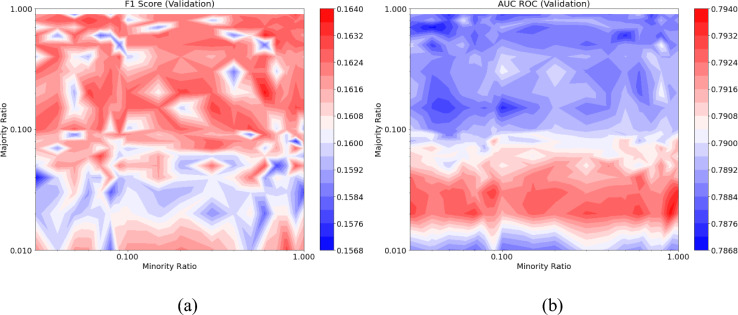
(**a**) F1-score; and (**b**) AUC–ROC; of the prediction model with combination of under-sampling, over-sampling, and class weighting.

In the ultimate scenario, a combination of under-sampling and over-sampling was applied to different extents, followed by class weighting to achieve a balance between the minority and majority classes. Figure 13a showcases the F1-score of the RF model under varying minority and majority ratios when predicting failures in the validation set. The highest F1-scores are attained at majority ratios surpassing 0.1. In other words, predictions suffer when under-sampling the majority class to a ratio below 0.1. Figure 13b illustrates the AUC–ROC for the validation set across different minority and majority ratios, when utilising RF model. In this instance, selecting a majority ratio below 0.1 is essential to achieve higher AUC–ROC values, indicating that a significant under-sampling of the majority class is necessary for optimal performance. Similar results for F1-score and AUC–ROC metrics were achieved when using an SVM model.

**Figure 14:**
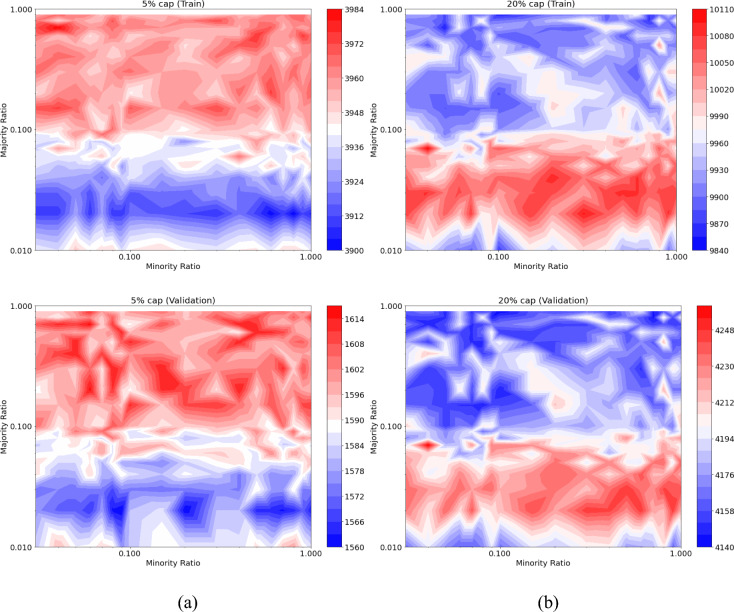
Number of failures captured by RF model when replacing (**a**) 5%; and (**b**) 20%; of pipes, with combination of under-sampling, over-sampling, and class weighting.

Figure 14a presents the number of captured failures when replacing 5% of pipes in both the train and validation sets of RF model. In both datasets, achieving improved failure predictions necessitates a majority ratio higher than 0.1. Similarly, Fig. 14b depicts the same for 20% pipe replacement, where higher failure predictions are observed when the majority ratio is below 0.1. In both cases, the minority ratio exerts limited influence on the outcomes, whereas the majority ratio significantly impacts the results. Conversely, when combining under-sampling and over-sampling, under-sampling the majority class emerges as a crucial factor affecting the number of predicted failures. Similar results for 5% and 20% pipe replacements were achieved when building an SVM model.

**Figure 15:**
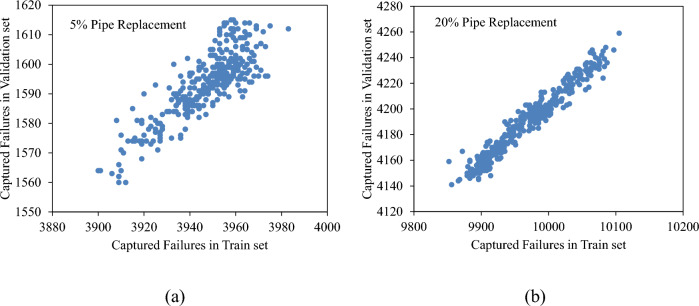
Number of failures captured in validation set vs. train set, when replacing (**a**) 5%; and (**b**) 20%; of pipes, with combination of under-sampling, over-sampling, and class weighting.

This scenario generates numerous combinations of minority ratios and majority ratios. To identify the optimal pair for predicting unseen data, it is crucial to derive insights from the training set. Accordingly, the number of predicted failures in the validation set is plotted against that in the training set. Figure 15 illustrates this graph for 5% and 20% pipe replacements. In both instances, the pairs of minority and majority ratios that resulted in higher predictions in the training set also yielded elevated predictions in the validation set. This implies that suitable values for the minority and majority ratios can be discerned from the training set and applied to predict failures in the validation set.

## Conclusion and remarks

This paper explores various strategies for handling imbalanced class data, focusing on three primary approaches: under-sampling, over-sampling, and class weighting. These methods aim to address the imbalance-ness in datasets by adjusting the representation of minority and majority classes. Under-sampling involves reducing the data in the majority class, over-sampling entails increasing the data in the minority class, and class weighting assigns unequal weights to classes based on their counts to balance their influence in training a ML model.

This study applied these approaches to develop a pipe failure prediction model in a WDN characterised by extreme imbalance class issue. Evaluation metrics, specifically F1-score and AUC–ROC, are chosen to assess the performance of the prediction models.

Under-sampling with various ratios for the majority class revealed that a ratio above the balance point yielded the highest F1-score. Over-sampling the minority class at ratios below the balance point resulted in the highest F1-score. Employing different class weights during training and prediction demonstrated that the best F1-score was achieved with a weight much lower than the balance.

Combining under-sampling and over-sampling to the same ratio for both majority and minority classes did not yield superior results compared to previous scenarios. However, a more effective prediction model emerged when over-sampling the minority class and under-sampling the majority class to different ratios, followed by applying class weights to balance the data. Despite the majority ratio, the results were not sensitive to minority ratio, suggesting that under-sampling has stronger effect on the prediction capability of the models, comparing with over-sampling.

A specific pipe replacement scenario was considered, where a 5% replacement ratio represents tactical asset maintenance in a WDN. Under-sampling yielded the highest number of predicted failures at a ratio exceeding the balance, while over-sampling was most effective at a ratio below the balance. Class weighting achieved the best results with a weight lower than the balance. Similarly, for a 20% pipe replacement scenario related to strategic asset maintenance, treating imbalanced class data led to 25–28% increase in predicted failures, in various scenarios. However, beyond the balance point, further under-sampling, over-sampling, or weighting did not significantly improve results, demonstrating that balancing the weights or number of instances leads to near-optimal results.

The combined application of all three approaches indicates that the minority ratio has a limited impact on prediction results, whereas changes in the majority ratio significantly influence outcomes. This sensitivity is attributed to the loss of valuable information through under-sampling. In tactical asset maintenance, majority ratios exceeding 0.1 provided better results, while in strategic asset maintenance, ratios below 0.1 led to improved predictions. Despite the benefits of balancing the dataset with under-sampling, caution is advised, as predictors are sensitive to this approach.

Although comparing the machine learning prediction models was not the primary focus of this research, the results indicate that a RF model slightly outperforms the SVM in predicting pipe failures in this case study. Practically, the optimal ratio for under-sampling, over-sampling, and class weighting can be determined by analysing the results in the training set. The study's findings provide valuable insights for asset managers, aiding in identifying pipes with a high likelihood of failures and facilitating preventive pipe replacement to mitigate bursts and breaks in a WDN, particularly in cases of limited failure data.

## Data Availability

Some or all data, models, or code generated or used during the study are proprietary or confidential in nature and may only be provided with restrictions. All case study data is owned by the utility company and is subject to a non-disclosure agreement (NDA), thereby limiting its availability for public dissemination. Requests for non-commercial usage of the scripts will be evaluated on a case-by-case basis, by contacting the corresponding author at m.latifi@exeter.ac.uk.
